# Effect of Smartphone-Based Telemonitored Exercise Rehabilitation among Patients with Coronary Heart Disease

**DOI:** 10.1007/s12265-019-09938-6

**Published:** 2019-12-09

**Authors:** Yanxin Song, Chuan Ren, Ping Liu, Liyuan Tao, Wei Zhao, Wei Gao

**Affiliations:** 1 Department of Cardiology and Institute of Vascular Medicine, Peking University Third Hospital, NHC Key Laboratory of Cardiovascular Molecular Biology and Regulatory Peptides, Key Laboratory of Molecular Cardiovascular Science, Ministry of Education, Beijing Key Laboratory of Cardiovascular Receptors Research, Beijing, 100191 China; 2 Research Center of Clinical Epidemiology, Peking University Third Hospital, Beijing, 100191 China; 3 Physical Examination Center, Peking University Third Hospital, Beijing, 100191 China

**Keywords:** Coronary heart disease, Telemonitored exercise rehabilitation, Exercise tolerance, Compliance

## Abstract

The aim of this study was to investigate the effects of telemonitored exercise rehabilitation on patients with coronary heart disease (CHD) in China. Ninety-six patients with stable CHD were included and analyzed (48 in telemonitored group and 48 in control group). All patients received routine follow-up, and patients in telemonitored group participated in smartphone-based telemonitored cardiac rehabilitation. Patients’ demographic information, medical history, diagnosis and treatment of CHD, and laboratory results were collected. The difference of cardiopulmonary exercise testing (CPET), blood test, and echocardiographic parameters; exercise habits; control rate of blood lipid and blood glucose; and incidence of adverse events between the two groups during 6 months of follow-up was analyzed. After intervention, the subjects in the telemonitored group performed significantly better in VO_2peak_, exercise compliance, and some other parameters than those in the control group. Telemonitored exercise rehabilitation is an effective rehabilitation mode for CHD patients in China.

## Introduction

In recent years, with the change of lifestyle, the prevalence of CHD has increased rapidly, which has become a serious public health problem. Cardiac rehabilitation, as an important intervention measure, is of great significance to improving the clinical outcomes of CHD patients [1]. However, patients’ participation in hospital-based cardiac rehabilitation is subject to many factors such as traffic and work [2]. Additionally, in a developing country like China, cardiac rehabilitation is not well developed and applied, because of unbalanced development of economy and technology among regions [3]. Therefore, it is an important task to improve the rehabilitation participation rate of patients in clinical practice.

Scientific and technological development enables the application and popularization of electronic products such as smartphones. In order to make full use of achievements of scientific and technological development and improve the efficiency of rehabilitation, some clinical workers have proposed and carried out home-based telemonitored cardiac rehabilitation with support of internet technology [4–6]. With the advantages of flexibility in location and time, this scheme may help solve the shortcomings of conventional cardiac rehabilitation [7]. Many studies abroad have proved the feasibility and effect of telemonitored cardiac rehabilitation [8–10]. But in China, telemonitored cardiac rehabilitation program was not yet mature; there were few studies on the acceptance and compliance to telemonitored rehabilitation of patients with CHD, or on the effect of telemonitored rehabilitation to these people. This study aimed to evaluate the application and effect of smartphone-based telemonitored exercise rehabilitation among CHD patients in China.

## Methods

### Study Design and Subject

This study was a single-center, prospective, randomized, controlled clinical trial. Patients who were enrolled in this study signed informed consent; then, they were randomly divided into telemonitored group (group A) and control group (group B) at the ratio of 1:1. All patients received cardiopulmonary exercise testing (CPET). After that, all of them were given exercise prescription according to their results of CPET. Exercise prescription was set according to their anaerobic threshold (AT). Exercise intensity was determined based on heart rates (HR). The target HR during exercise was set as heart rate@AT (HR@AT) ± 5 bpm. The exercise type was walking. Exercise frequency was 3–5 times per week, with each exercise duration of 30 min and 5–10 min of warm-up and relaxation before and after exercise [11]. Both groups received routine discharge education and outpatient follow-up which included advice to exercise regularly. This study passed the review by the Ethics Committee of Peking University Third Hospital (approval number 2014203), and followed the CONSORT reporting standard.

Inclusion Criteria

Inclusion criteria were the following: (1) age≤ 75 years old; (2) diagnosed as stable CHD by coronary angiography; (3) without physical or mental disorders affecting exercise; (4) skillful in using software such as WeChat and telemonitoring software.

Exclusion Criteria

Exclusion criteria were as follows: (1) with congestive heart failure of class III–IV under New York Heart Association (NYHA) Classification or class III–IV under Killip Classification; (2) accompanied by severe diseases of other systems, such as HIV, malignant tumors, severe primary liver, and kidney diseases; (3) coexisting with clinical conditions with life expectancy of less than 6 months, or unable to complete the follow-up; (4) refused to sign the informed consent or unable to exercise or not willing to cooperate; (5) participated in other interventional clinical studies at the time of enrollment or within 30 days before enrollment.

### Intervention

Researchers helped install telemonitoring software (MEMRS-CRS, developed by Medicus) on patients’ smartphones in group A, distributed heart rate belts (Suunto, provided by Medicus) for monitoring patients’ HR, and instructed patients in wearing heart rate belts and using the software. Medical staff monitored patients’ exercise frequency, blood pressure (BP) and HR before and after exercise, investigated fatigue degree after exercise using the Borg Rating of Perceived Exertion Scale (RPE 6-20) [12] at the Medicus monitoring device computer terminal, and communicated with patients weekly through text messaging (WeChat or other applications) and telephone call. Feedback was provided on patients’ exercise status (including the frequency, intensity and time of exercise, and blood pressure and HR response before and after exercise), questions about device usage, and exercise prescriptions. The frequency of feedback was usually once a week. Researchers checked the information on the website every day commonly. If patients have questions, they would answer them in time. The feedback was based on cognition and behavior theory and was personalized. For both of the two arms, the participants did not accept any in-hospital cardiac rehabilitation.

### Baseline Information

Patients’ demographic information was collected, including gender, age, height, weight, occupation, and education; exercise habits; application of smartphones; past medical history; diagnosis and treatment of CHD; and blood test, echocardiography, and CPET results.

### Follow-up

Patients’ CPET and echocardiography parameters, exercise habits, blood test information, including blood cell count, liver function, kidney function, blood lipid, and blood glucose, and the incidence of clinical events and adverse events were collected after 6 months of intervention.

### Endpoints

The primary endpoint was the difference between the two groups in changes of exercise tolerance, represented by VO_2peak_ and other CPET parameters during follow-up. Secondary endpoints included the difference of exercise habits (According to the American College of Sports Medicine (ACSM) 10th edition of “‘ACSM’ Guidelines for Exercise Testing and Prescription,” exercise 3–5 times a week, each exercise time (> 30 min) means having exercise habits, and the rest for no exercise [11]), blood test and echocardiographic parameters, control rate of blood lipid and blood glucose, and incidence of adverse events after 6 months of follow-up.

### Statistical Methods

#### Sample Size

According to relevant literature, VO_2peak_ increased by 14.3% in the telemonitored rehabilitation group and 0% in the conventional rehabilitation group [13]. Using bilateral test with an *α* of 0.05 and *β* of 0.2 (efficacy of 80%) and a case distribution ratio of 1:1 in the two groups, it was estimated that 48 patients were needed in each group. Considering 10% dropouts and other factors, this study needed to enroll a total of 106 patients, including 53 in the experimental group and 53 in the control group.

#### Data Analysis

SPSS software version 20.0 (IBM, Chicago, IL, USA) was used for data analysis. Quantitative variables were expressed as mean ± standard deviation (*x* ± S) in normal distributions, and as median and interquartile range in cases that were not normally distributed. Qualitative variables were expressed as percentages. Chi-square test, independent sample *t* test, and repeated measures ANOVA were used to compare variables between the two groups. Chi-square test and logistics regression were used for correlation analysis. The level of significance was defined as *P* = 0.05.

## Results

### Baseline Data

**Fig. 1:**
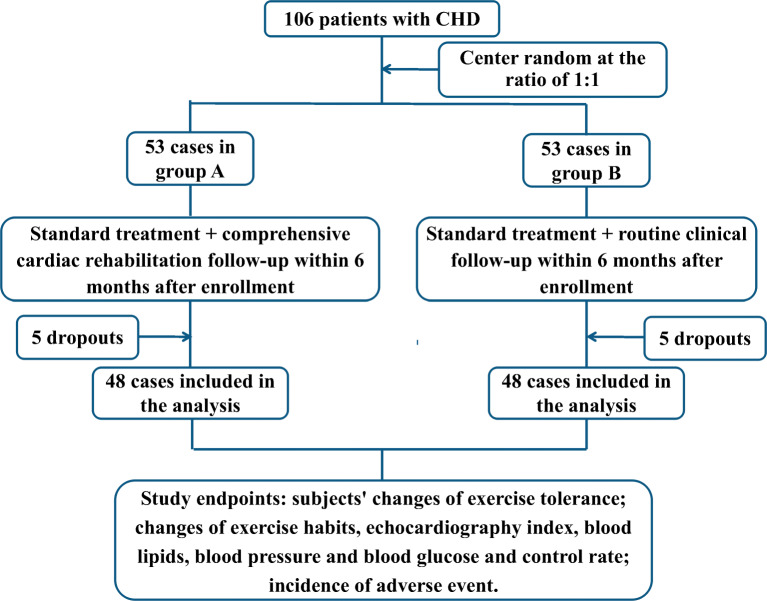
Flow chart of the study

One hundred thirty-three CHD patients who visited the Department of Cardiology, Peking University Third Hospital, from September 2017 to September 2018 were screened for the purpose of this study, of which 13.5% refused to participate and 6.8% fulfilled exclusion criteria, and 106 patients were selected eventually. Of those 106 CHD patients, a total of 96 (48 in group A and 48 in group B) completed the follow-up. None of the patients had atrial fibrillation. In group A, the average age of patients was 54.17 ± 8.76 years old, and 43 patients (89.60%) were male. For the five dropouts in group A, one failed to master the application of mobile phone monitoring software; one withdrew due to work reasons; three failed to continuously wear monitoring device. In group B, the average age of patients was 54.83 ± 9.13 years old, and 40 patients (83.33%) were male. For the five dropouts in group B, two withdrew due to work or family reasons; three refused to visit the hospital for follow-up after 6 months. Figure 1 presents the flow chart of the study.

There was no significant difference in baseline data between the two groups (*P* > 0.05), as shown in Tables 1 and 2.

**Table 1:** Comparison between the two groups in medical history

Items	Group A number (%)/mean ± S	Group B number (%)/mean ± S	*χ*^2^/*t*	*P* value
Male	43(89.60)	40(83.33)	0.801	0.371
Age	54.17 ± 8.76	54.83 ± 9.13	− 0.365	0.716
Height (cm)	170.50 ± 6.60	170.10 ± 6.36	0.299	0.765
Weight (kg)	77.84 ± 12.45	75.93 ± 13.51	0.854	0.473
High school or above	27(56.2)	19(39.6)	2.671	0.102
Smoking	32(66.7)	26(54.2)	1.568	0.210
Myocardial infarction	26(54.2)	22(45.8)	0.667	0.414
Hypertension	29(60.4)	37(77.1)	3.103	0.078
Hyperlipidemia	37(77.1)	31(64.6)	1.815	0.178
Diabetes mellitus	26(54.2)	20(41.7)	1.503	0.220
Cerebral infarction	2(4.2)	0(0)	2.403	0.475
Family history	22(45.8)	18(37.5)	0.686	0.408
Exercise habits	36(75.0)	34(70.8)	0.211	0.646
Numbers of lesion vessel	2.06 ± 0.90	1.85 ± 0.92	1.124	0.264
Complete revascularization	23(47.9)	32(66.7)	3.448	0.063
Statin	44(91.7)	46(95.8)	0.178	0.673
Βeta-blocker	28(58.3)	28(58.3)	0.000	1.000
Aspirin	35(72.9)	34(70.8)	0.052	0.820
Clopidogrel	22(45.8)	28(58.3)	1.503	0.220
ACEI/ARB	15(31.2)	17(35.4)	0.188	0.665

**Table 2:** Comparison of examination results between the two groups at baseline

Items	Group A, mean ± S	Group B, mean ± S	*t* test	*P* value
SBP@baseline (mmHg)	125.47 ± 16.19	128.26 ± 13.89	− 0.813	0.419
DBP@baseline (mmHg)	76.56 ± 12.35	77.74 ± 10.81	− 0.450	0.654
HGB(g/L)	148.14 ± 11.82	142.28 ± 17.11	1.768	0.081
Blood glucose (mmol/L)	6.45 ± 1.58	6.91 ± 1.82	− 1.276	0.205
TC(mmol/L)	3.42 ± 0.93	3.54 ± 0.94	− 0.631	0.530
TG (mmol/L)	1.68 ± 1.07	1.85 ± 1.02	− 0.762	− 0.448
LDL-C (mmol/L)	1.96 ± 0.69	2.07 ± 0.83	− 0.678	0.500
HDL-C (mmol/L)	1.04 ± 0.27	0.98 ± 0.20	1.051	0.296
ALT (U/L)	28.58 ± 12.78	25.35 ± 16.10	1.059	0.293
AST (U/L)	25.16 ± 20.22	21.57 ± 11.69	1.040	0.301
Scr (μmol/L)	88.12 ± 15.47	87.66 ± 15.41	0.131	0.896
UA (μmol/L)	334.63 ± 123.42	323.64 ± 138.70	0.375	0.709
LAD (mm)	34.70 ± 6.21	35.14 ± 5.74	− 0.344	0.731
LAA (cm^2^)	18.92 ± 3.07	19.11 ± 3.11	− 0.285	0.777
LVEDd (mm)	49.99 ± 5.43	49.43 ± 4.92	0.503	0.616
LVEDs (mm)	32.22 ± 7.00	31.03 ± 6.32	0.832	0.408
LAP (mmHg)	9.61 ± 2.65	10.32 ± 3.50	− 1.072	0.287
EF (%)	63.98 ± 10.70	66.25 ± 9.06	− 1.075	0.285
VO_2peak_ (mL/kg/min)	20.40 ± 4.57	18.83 ± 3.98	1.771	0.080
VO_2peak_ pred%	67.45 ± 13.48	64.02 ± 13.36	1.230	0.222
HR_peak_ (bpm)	130.77 ± 16.52	123.93 ± 17.5	1.946	0.055
VO_2_/HR@VO_2peak_	12.09 ± 2.76	11.62 ± 2.86	0.798	0.427
SBP@VO_2peak_ (mmHg)	170.29 ± 32.36	174.20 ± 30.96	− 0.591	0.556
DBP@VO_2peak_ (mmHg)	77.42 ± 10.34	76.16 ± 13.50	0.504	0.615
AT (mL/kg/min)	12.35 ± 2.75	11.60 ± 2.47	1.398	0.165
HR@AT (bpm)	103.15 ± 12.78	100.75 ± 11.67	0.933	0.353
VO_2_/HR@AT	9.29 ± 2.01	8.74 ± 2.27	1.199	0.549
VE/VCO_2_@AT	28.50 ± 3.63	29.89 ± 3.71	− 1.810	0.074
VE/VCO_2_ slope	28.41 ± 4.98	29.87 ± 4.39	− 1.494	0.139
OUES	1679.85 ± 419.08	1578.35 ± 368.37	1.234	0.221

Quantitative variables are expressed as mean ± standard deviation in normal distributions. *SBP*, systolic blood pressure; *DBG*, diastolic blood pressure; *HGB*, hemoglobin; *TC*, total cholesterol; *TG*, triglyceride; *LDL-C*, low-density lipoprotein cholesterol; *HDL-C*, high-density lipoprotein cholesterol; *Scr*, serum creatinine; *UA*, uric acid; *LAD*, left atrial diameter; *LAA*, left atrial area. *LVEDd*, end-diastolic diameter of left ventricle; *LVEDs*, end-systolic diameter of left ventricle; *LAP*, left atrial pressure; *EF*, ejection fraction; *VO*_*2peak*_, peak oxygen uptake: *VO*_*2peak*_ *pred%*, percentage of predicted peak oxygen uptake; *HR*_*peak*_, peak heart rate; *VO*_*2*_*/HR*, oxygen pulse; *AT*, anaerobic threshold; *HR@AT*, heart rate at anaerobic threshold; *VE/VCO*_*2*_, ventilatory equivalent for carbon dioxide; *VE/VCO*_*2*_ *slope*, the relationship between change in V̇E and V̇CO_2_ during incremental exercise; *OUES*, oxygen uptake efficiency slope

Qualitative variables are expressed as number (percentage). Quantitative variables are expressed as mean ± standard deviation in normal distributions. *ACEI*, angiotensin-converting enzyme inhibitor; *ARB*, angiotensin receptor blocker

### Rehabilitation Effectiveness

The exercise frequency of patients in group A was 5.1 ± 0.6 times a week; each time was 31.4 ± 4.5 min. The duration of HR reaching the target HR during exercise accounted for (94.2 ± 6.4)% of the total exercise time. In addition, 91.7% (44/48) of the participants in the intervention group were satisfied with the content and frequency of feedback; others were not completely satisfied with the feedback because they were not proficient in uploading information even with guidance of researchers.

#### Improvement in exercise tolerance

**Fig. 2:**
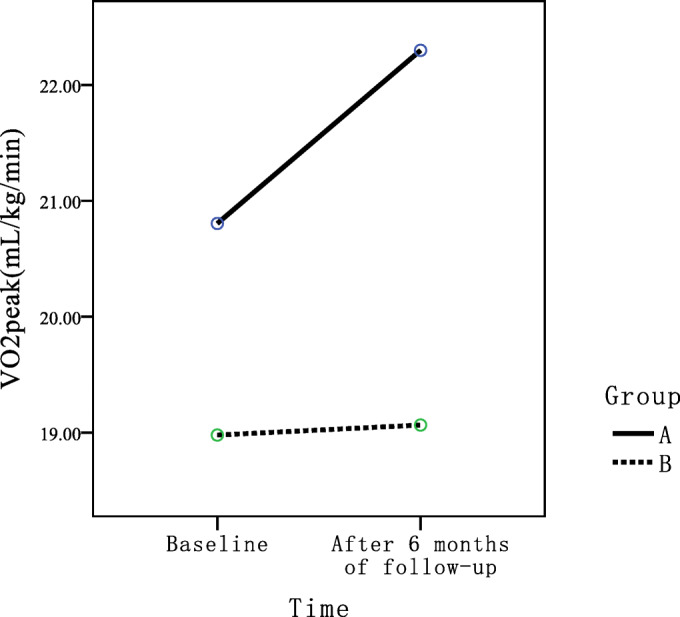
Interaction profile of VO_2peak_

CPET parameters, mainly VO_2peak_, were used to measure patients’ exercise tolerance in this study. The VO_2peak_ values of the two groups of patients before and after intervention were analyzed by two-factor and two-level repeated measures ANOVA. Results showed that the main effect of intervention was statistically significant (*P* = 0.007), indicating that there was significant difference in VO_2peak_ between the two groups regardless of the time factor. The main effect of time was also statistically significant (*P* = 0.033), indicating that VO_2peak_ values of patients changed over time regardless of the intervention factor. The interaction between time effect and intervention effect was not statistically significant (*P* = 0.056) (Fig. 2).

The VO_2peak_ after intervention in group A and group B was 22.29 ± 4.79 (mL/kg/min) and 19.07 ± 5.33 (mL/kg/min), respectively (*t* = 3.012, *P* = 0.003), indicating that there were statistically significant differences in VO_2peak_ between the two groups after intervention.

During the follow-up period, other indicators that could reflect patients’ exercise tolerance and cardiopulmonary function with statistical differences included VO_2peak_ pred%, HR_peak_, AT, VE/VCO_2_@AT, VE/VCO_2_ slope, and OUES (Table 3).

**Table 3:** Indicators with statistically significant differences between the two groups after 6 months

Indicator	Group A	Group B	*t* test	*P* value
VO_2peak_ pred%	72.05 ± 14.98	64.66 ± 17.04	2.160	0.034
HR_peak_ (bpm)	135.60 ± 16.38	121.61 ± 15.22	4.171	< 0.001
AT (mL/kg/min)	13.42 ± 3.95	11.64 ± 3.46	2.253	0.027
VE/VCO_2_@AT	27.24 ± 2.87	29.52 ± 3.71	− 3.161	0.002
VE/VCO_2_ slope	27.78 ± 3.91	29.52 ± 3.71	− 1.864	0.002
OUES	1777.38 ± 416.50	1565.99 ± 381.52	2.495	0.014

Quantitative variables are expressed as mean ± standard deviation in normal distributions; *VO*_*2peak*_ *pred%*, percentage of predicted peak oxygen uptake; *HR*_*peak*_, peak heart rate; *AT*, anaerobic threshold; *VE/VCO*_*2*_*@AT*, ventilatory equivalent for carbon dioxide at anaerobic threshold; *VE/VCO*_*2*_ *slope*, the relationship between change in V̇E and V̇CO_2_ during incremental exercise; *OUES*, oxygen uptake efficiency slope

#### Improvement in Exercise Habits

Changes of patients’ exercise habits from baseline to 6 months are presented in Table 4. In each group, 9 patients (18.8%) did not have exercise habit when enrolled, but insisted on exercise after 6 months. Six patients (12.5%) in group B had exercise habits when enrolled, but failed to keep exercise after 6 months. Other patients had no change in exercise habits. There was a statistically significant difference in changes of exercise habits between the two groups (*P* = 0.020).

**Table 4:** Changes of patients’ exercise habits before and after intervention in the two groups

Group	Whether patients have exercise habits before and after intervention				*χ*^2^	*P* value
	No/no, number (%)	No/yes, number (%)	Yes/no, number (%)	Yes/yes, number (%)	9.826	0.020
A	3 (6.2)	9 (18.8)	0 (0.0)	36 (75.0)		
B	5 (10.4)	9 (18.8)	6 (12.5)	28 (58.3)		

Results of univariate and multivariate analyses of patients’ exercise habits during the follow-up period are shown in Table 5. Whether one had exercise habit during the follow-up period only had statistical correlation with study group and baseline exercise habits, but no statistical correlation with other factors.

**Table 5:** Multivariate analysis of patients’ exercise habits after intervention

Variables included	B	S.E.	Wals	*P*	OR	95% confidence interval
Under telemonitored monitoring	1.531	0.712	4.622	0.032	4.624	1.145~18.677
With exercise habits at baseline	1.591	0.627	6.429	0.011	4.907	1.435~16.784

#### Others

During the follow-up period, there were no statistically significant differences in biochemical and echocardiographic parameters between the two groups. There was no significant change in the control rate of blood lipid and blood glucose after 6 months of follow-up. No serious complications or adverse events occurred during follow-up.

## Discussion

Currently, cardiac rehabilitation for patients with CHD in China, especially rehabilitation in the out-of-hospital setting, is still not perfect. Limitations in various aspects lead to poor participation of patients in rehabilitation, especially for the elderly. In view of this, we designed this research program and made full use of widely used smartphones and telemonitoring devices to extend the cardiac rehabilitation setting from hospitals to homes [14]. Combining HR monitoring during exercise with personalized feedback from patients, this study contributes to the domestic research system of smartphone-based, long-term intervention for patients with stable CHD. Therefore, it is a practical and valuable study for patients with stable CHD.

### Improvement in Exercise Tolerance

Results of this study showed that patients in the intervention group had more significant improvement in exercise tolerance (represented by VO_2peak_), compared with those in the control group. In this study, we used CPET to evaluate patients’ exercise tolerance. For CPET, pulmonary gas exchange is measured by recording and analyzing exhaled gas of patients so as to further assess exercise-related physiology characteristics of cardiovascular, respiratory, musculoskeletal, and cellular oxidative systems [15, 16]. It provides patients with comprehensive and accurate information about their exercise tolerance and exercise limitation. VO_2peak_ is the VO_2_ value the tester attained with maximum effort in the incremental load exercise test and is significantly correlated with individual exercise tolerance and disease prognosis [15–18]. By analyzing changes of VO_2peak_ in the two groups before and after intervention, this study found statistically significant difference in VO_2peak_ between the two groups after 6 months of intervention and changes of VO_2peak_ with time in both groups. It must be noted that both study arms indicated a tendency towards improvement regardless of intervention; however, the improvement was more obvious in group A. Frederix’s study [19] and Duscha’s study [20] both found that VO_2peak_ improved in the intervention group after telemonitored rehabilitation, while this variable did not improved in the conventional rehabilitation group. However, Kraal et al. [8] showed that both the telemonitored monitoring group and the conventional rehabilitation group had improvement in exercise tolerance after regular rehabilitation. All of those studies demonstrated the effects of telemonitored rehabilitation were similar to or even better than those of conventional rehabilitation. In this study, HR changes of patients in intervention group during exercise were monitored in real time by smartphones and electronic monitoring devices, and medical staff regularly gave feedback to patients on their exercise and physical parameters with the aid of computer monitoring terminal, thereby improved patients’ understanding of their own physical condition and also external support received during rehabilitation process. It may be an important reason for patients’ improvement in rehabilitation compliance with exercise intensity, time, and frequency, and then promoted the improvement of patients’ exercise tolerance.

Additionally, it was found that VO_2peak_ pred%, HR_peak_, AT, VO_2_/HR@AT, VE/VCO_2_@AT, VE/VCO_2_ slope, and OUES were significantly better in the intervention group than in the control group after 6 months of intervention (*P* < 0.05). In a study of 142 patients with CHD, Popovic et al. found significant improvement of patients in VO_2peak_, VO_2_/HR@VO_2peak_, VE/VCO_2_ slope, and OUES after 3 to 6 months of cardiac rehabilitation [21]. Although changes of specific indicators are not exactly the same in those two studies, both of them demonstrated certain effects of monitored cardiac rehabilitation. Previous studies have confirmed the practical significance of improvement in the above indicators [22, 23]. As AT, VO_2_/HR@AT, VE/VCO_2_, and OUES are all indicators for submaximal exercise, this study is of great practical significance for patients who can only perform submaximal exercise tests.

### Improvement in Exercise Habits

Results showed that there were statistically significant differences in exercise habits between the two groups during the follow-up period (*P* = 0.020). During follow-up, the proportion of patients with exercise habits in telemonitored group was 93.8%, significantly higher than that in the conventional follow-up group (77.1%) (*P* = 0.021). But this proportion was still lower compared with the study results by Piotrowicz, which showed that up to 99.2% of patients were completely and partially compliant with telemonitored rehabilitation [24]. However, the period of study by Piotrowicz (4 weeks) was different from that of this study. Therefore, it could not fully indicate that the rehabilitation compliance of patients in the former study was better than our research. In this study, whether one had exercise habit or not during the follow-up period only had statistical correlation with intervention and baseline exercise habits (*P* < 0.05). It indicated that telemonitoring could improve patients’ compliance with exercise rehabilitation and those who have had exercise habits before the intervention were more likely to maintain exercise habits. Similar results have been obtained in foreign studies. In a study of 53 patients with chronic stable heart failure, Hwang [2] showed that the compliance rates of telemonitored rehabilitation group and conventional rehabilitation group were 71% and 30%, respectively. In a study of rehabilitation among patients with myocardial infarction, Varnfield et al. [25] showed that the participation rate of patients in the telemonitored rehabilitation group was up to 94%. Timely feedback from medical staff may promote the maintenance and development of patients’ exercise habits.

### Innovation in Telemonitored Cardiac Rehabilitation

During the implementation of this study, patients in group A wore heart rate belts while exercising. Through observing HR changes in real time by mobile phones, they could control the exercise intensity. Technological advance has made it possible for patients under telemonitoring to obtain reliable data support during exercise [26]. In addition, CPET, a gold standard with convenient and safe operation, was adopted for assessing patients’ exercise tolerance and developing exercise prescriptions [11]. With an intervention period of 6 months, which was much longer than the intervention period of other telemonitored intervention studies nationwide [27, 28], this study can provide reference for telemonitored cardiac rehabilitation practice in China.

### Limitations and Prospects

This study was limited by smartphones using and communicational methods. What’s more, patients using the mobile devices may be more comfortable with using mobile devices and also more engage in their own healthcare. Therefore, we need to further explore a more widely applicable telemonitored intervention mode. In addition, it must be noted that researchers’ feedback to patients would also have a certain impact on patients’ exercise habits. Because of absenting of subsequent follow-up after half-year’s intervention, long-term effects of telemonitored rehabilitation cannot be determined. Further large-scale, multi-center studies involving different diseases and hospitals of different levels and in different regions are still needed to further clarify the value of telemonitored cardiac rehabilitation. In this study, we did not conduct an explicit investigation on the economic cost of the participants, so we had no clear knowledge about the cost-effectiveness of the telemonitored rehabilitation program. Additionally, if telemonitored rehabilitation is widely used in clinic in the future, considering the safety of remote monitoring equipment, protecting patients’ privacy is indeed an important part of clinical work.

## Abbreviations

CHD: Coronary heart disease
CPET: Cardiopulmonary exercise testing
HR: Heart rate
BP: Blood pressure
VO_2peak_: Peak oxygen uptake
VO_2peak_ pred%: Percentage of predicted peak oxygen uptake
HR_peak_: Peak heart rate
AT: Anaerobic threshold
HR@AT: Heart rate at anaerobic threshold
RPE: Rating of perceived exertion
VO_2_/HR: Oxygen pulse
VE/VCO_2_: Ventilatory equivalent for carbon dioxide
VE/VCO_2_ slope: The relationship between change in V̇E and V̇CO_2_ during incremental exercise
OUES: Oxygen uptake efficiency slope
